# The retrospective study of perioperative application of dexamethasone and furosemide for postoperative anti-inflammation in patients undergoing percutaneous nephrolithotomy

**DOI:** 10.1007/s11255-020-02718-1

**Published:** 2021-01-07

**Authors:** Taiguo Qi, Xia Qi, Xiude Chen, Xunbo Jin

**Affiliations:** 1 Department of Urology, Cheeloo College of Medicine, Shandong Provincial Hospital, Shandong University, No. 324 Jingwu Road, Huaiyin District, Jinan, 250021 Shandong China; 2 Department of Urology, The Second Affiliated Hospital Affiliated to Shandong First Medical University, Taian, Shandong China

**Keywords:** Percutaneous nephrolithotomy, Dexamethasone, Furosemide

## Abstract

**Objectives:**

To investigate whether the perioperatively combined application of dexamethasone and furosemide could alleviate the inflammation in patients undergoing percutaneous nephrolithotomy (PCNL).

**Patients and methods:**

147 patients undergoing PCNL between November 2018 and October 2019 were enrolled in the study. 77 patients accepted a single dose of dexamethasone and furosemide administration (EXP group, *n* = 77), and 70 patients did not (CON group, *n* = 70). Demographic and perioperative data, inflammatory markers including interleukin-6 (IL-6) and procalcitonin (PCT), and clinical outcomes were compared between the two groups.

**Results:**

Compared with the CON group, the incidence rate of urosepsis of the EXP group were significantly lower (11.69% vs. 24.29%, *p* = 0.046). 3 patients developed severe urosepsis in the EXP group, while 5 patients developed severe urosepsis in the CON group. Compared with those in the CON group, the patients with postoperative urosepsis in the EXP group showed lower serum levels of IL-6 at postoperative hour two (*p* = 0.045) and at postoperative day one (*p* = 0.031) and lower serum levels of PCT at postoperative day one (*p* = 0.015). There was a better clinical outcome of a shorter postoperative hospital stay (*p* = 0.015) in patients with postoperative urosepsis in the EXP group than in those in the CON group.

**Conclusion:**

The perioperatively combined application of dexamethasone and furosemide was beneficial for alleviating postoperative inflammatory reaction and caused a better clinical outcome of a shorter postoperative hospital stay.

## Introduction

Urolithiasis is a common urological disease and affects people’s health with a prevalence of 5–10% worldwide[1, 2]. The methods of urolithiasis were PCNL, ureteroscopic lithotripsy, ESWL, and open surgery. PCNL has been the standard treatment for large, multiple renal stones with a high stone-free rate of over 90% since its first introduction in 1976 [3–6]. The most common complication of PCNL is a postoperative infection, with an incidence rate of 21–31.2% [7], which can progress to potentially life-threatening urosepsis with an incidence rate of 0.3–4.7% ranging from SIRS to uroseptic shock [8–10]. Urosepsis is a critical situation with 30–40% mortality [11]. Several studies have shown some factors such as old age, female gender, diabetes mellitus, high stone burden and positive urine culture may predict incidences of postoperative urosepsis [12, 13], however, urosepsis is difficult to avoid and may cause serious clinical outcomes even if the preoperative urine culture is negative and prophylactic antibiotics are given [14–16]. Delay in diagnosis and treatment also causes more severer infection, which will prolong the postoperative hospital stays and increase mortality. Furthermore, there is a significantly stepwise increase in the mortality rate from SIRS to uroseptic shock. Thus, it is necessary to explore procedures that can alleviate infection-related complications and prevent the process of systemic infection. Dexamethasone, as one type of glucocorticoid, has a great anti-inflammatory effect, and furosemide has diuretic effect which may reduce the absorbing of bacteria and endotoxins. In the past clinical trials, we found the perioperative application of dexamethasone and furosemide might be helpful for anti-inflammation. The study was designed to test that and investigate a way that could alleviate postoperative inflammatory reactions in patients undergoing PCNL.

## Patients and methods

We reviewed consecutive patients of PCNL for renal calculi between November 2018 and October 2019 in our center. In total, 147 patients were ultimately recruited into our study for further analysis. Inclusion criteria were: (1) age ≥ 18 years, (2) preoperative values of PCT and IL-6 are normal, (3) a positive leukocyturia, as defined by a positive leukocyte esterase dipstick test, or pyuria, defined by the presence of more than five leukocytes per high-power field in a centrifuged sediment before PCNL, or at least one symptom of UTI (dysuria, urgency, frequency, perineal pain, flank pain or costovertebral tenderness). Exclusion criteria were: (1) age < 18 years, (2) preoperative fever within one week, (3) the data of PCT and IL-6 was incomplete, (4) postoperative transfusion or embolization, (5) a history of kidney transplantation, (6) polycystic kidney diseases, (7) hemodialysis or peritoneal dialysis, (8) history of tumors, blood disease or chemotherapy, and (9) preoperative steroid use. 3 patients in EXP group and 4 patients in CON group that required postoperative transfusion were excluded, 3 patients in EXP group and 2 patients in CON group with fever within 1 week were excluded, 2 patients in EXP group diagnosed with polycystic kidney disease were excluded, 1 patient in CON group diagnosed with systemic lupus erythematosus was excluded, and 4 patients in EXP group and 3 patients in CON group with incomplete data were excluded.

Among the 147 patients, 77 patients accepted dexamethasone and furosemide administration (EXP group, *n* = 77), and 70 patients did not (CON group, *n* = 70). In the EXP group, a single dose of dexamethasone 10 mg was intravenously injected when percutaneous access was established and lithotripsy started, and 5 min later, a single dose of furosemide 10 mg was also intravenously injected, and that was called “Double Ten Principle” by us.

All these 147 patients’ medical records were reviewed retrospectively. Renal stones were demonstrated by non-contrast CT before PCNL, the total stone burden was calculated by length × width × π × 0.25 [17], and patients were classified into two groups with the low (stone burden ≤ 353 mm2) or high (stone burden > 353 mm2) stone burden [8], and urine was tested before antibiotic treatment. The values of PCT and IL-6 levels were collected at preoperative day one, at postoperative hour two and at postoperative day one. The same surgeon performed all the surgeries adhering to the standard technique [18, 19]. The surgeries were considered completed when stones were not detected under the endoscope. Operative time was measured from the insertion of the ureteral catheter until suture. Vital signs were closely observed, and postoperative complications were documented. In our study, the same antibiotic was used prophylactically following the guidelines in patients preoperatively and the same type of antibiotics would be used when the infection was aggravated postoperatively.

SIRS was defined as occurrence of 2 or more of the followings: (1) body temperature > 38 °C or < 36 °C, (2) heart rate > 90 beats/min, (3) respiratory rate > 20 breaths/min or arterial carbon dioxide tension < 32 mmHg, (4) leucocyte count > 12 × 10^9^/L or < 4 × 10^9^/L. Severe urosepsis referred to SIRS complicated by organ dysfunction [20].

All patients were told the possibility of dexamethasone and furosemide administration perioperatively during preoperative conversation and accepted voluntarily. All patients were required to write informed consent for their data to be used for research purposes.

The normality of quantitative data was tested by Kolmogorov–Smirnov test. The Student’s *t* test, the *χ*^2^ test, Scatter Diagrams and Random Forest methods were used to analyze these data. It was considered statistically significant when the two-tailed *p* values < 0.05.

## Results

### (1) Clinical characteristics

Of all the 147 patients enrolled, 77 patients were classified into the EXP group, and the others were classified into the CON group. There were not significant differences in factors such as gender (*p* = 0.962), age (*p* = 0.426), diabetes (*p* = 0.079), hypertension (*p* = 0.502), hydronephrosis (*p* = 0.365), stone burden (*p* = 0.566), pyuria (*p* = 0.670), urine culture (*p* = 0.793), operative time (*p* = 0.201), preoperative serum levels of IL-6 (*p* = 0.111) and PCT (*p* = 0.717).

### (2) Clinical outcomes

In the EXP group, 9 patients progressed to urosepsis, while 19 patients progressed to urosepsis in the CON group, the incidence rates of urosepsis were significantly lower in the EXP group than in the CON group (11.69% vs. 24.29%, *p* = 0.046). 3 patients progressed to severe urosepsis in the EXP group, while 5 patients progressed to severe urosepsis in the CON group.

The EXP group showed a significantly shorter postoperative hospital stay (4.45 ± 1.561 vs. 5.09 ± 2.083, *p* = 0.041) compared with the CON group. When postoperative urosepsis occurred, patients in the EXP group showed a shorter postoperative hospital stay (5.11 ± 1.269 vs. 6.53 ± 1.328, *p* = 0.015). When postoperative urosepsis did not occur, the postoperative hospital stay of patients in EXP group was lower but not statistically significant (4.37 ± 1.583 vs. 4.62 ± 2.078, *p* = 0.445). The results are listed in Table 1.

**Table 1:** Clinical characteristics and outcomes

Patient characteristic	EXP group (*n* = 77)	CON group (*n* = 70)	*p* values
Gender (Male)	41	37	0.962
Age (years)	52.62 ± 11.73	54.16 ± 11.53	0.426
Diabetes	20	10	0.079
Hypertension	23	19	0.502
Hydronephrosis	63	53	0.365
Stone burden > 353mm^2^	23	24	0.566
Urine culture	27	26	0.793
Pyuria	48	46	0.670
Operative time(min)	81.04 ± 34.32	87.86 ± 29.58	0.201
postoperative hospital days	4.45 ± 1.56	5.09 ± 2.08	0.041
Urosepsis	5.11 ± 1.27	6.53 ± 1.33	0.015
Non-urosepsis	4.37 ± 1.58	4.62 ± 2.08	0.445
Urosepsis	9	17	0.046
SIRS	6	12	0.084
Severe urosepsis	3	5	0.615

### (3) PCT and IL-6

**Fig. 1:**
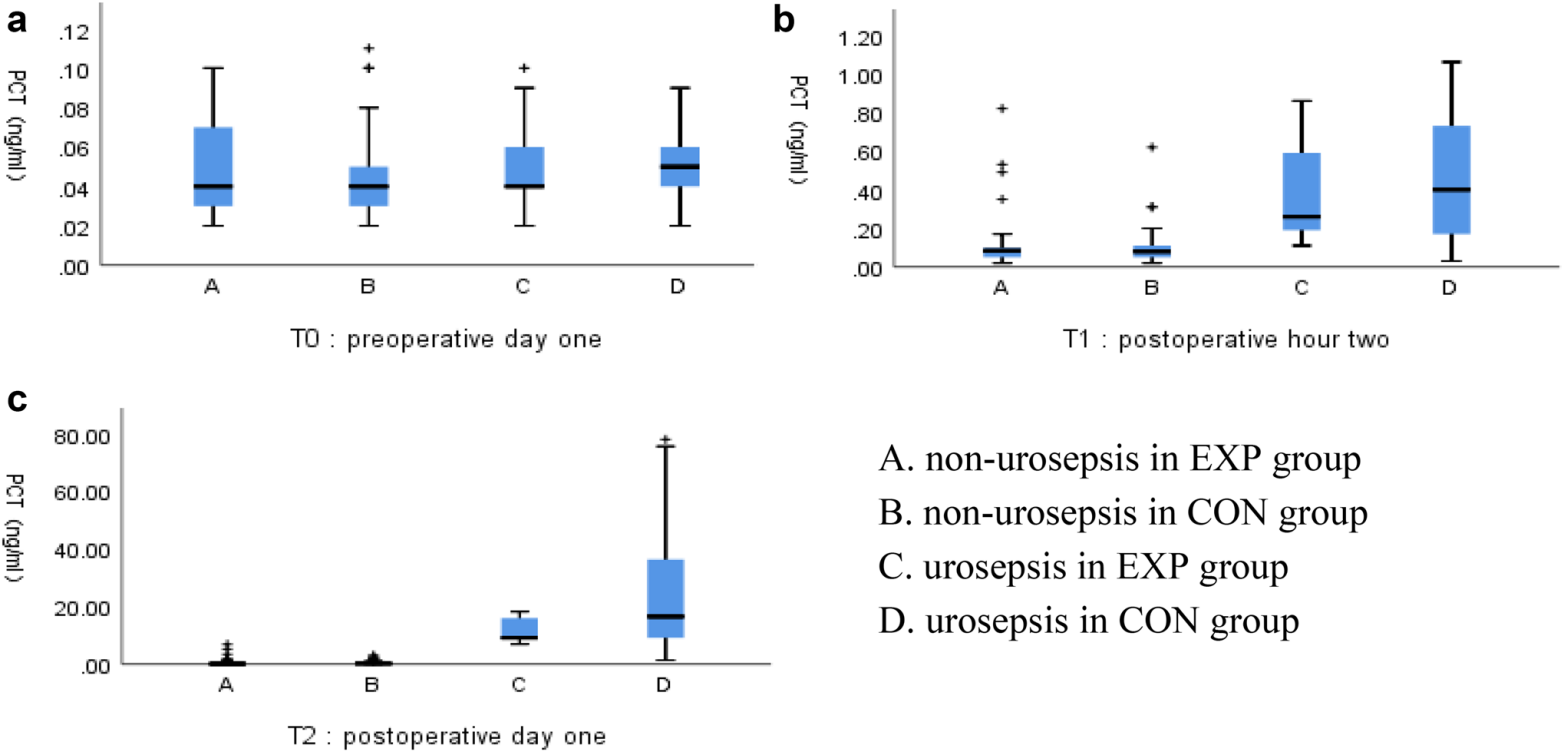
The expression of PCT in different groups (A non-urosepsis in EXP group, B non-urosepsis in CON group, C urosepsis in EXP group, D urosepsis in CON group), **a** the expression of PCT at preoperative day one, **b** the expression of PCT at postperative hour two, **c** the expression of PCT at postperative day one

At preoperative day one (T0), univariate analysis showed no significant differences in serum levels of PCT (*p* = 0.717) between the EXP group and the CON group. At postoperative hour two (T1), there were no significant differences in serum levels of PCT (*p* > 0.05) between the EXP group and CON group. At postoperative day one (T2), compared with the CON group, the EXP group showed a lower serum level of PCT (1.86 ± 4.12 vs. 6.85 ± 16.32, *p* = 0.015), the serum level of PCT was lower and statistically significant in patients with postoperative urosepsis (11.98 ± 4.52 vs. 26.57 ± 24.54, *p* = 0.029), and the differences between the two groups in patients with postoperative non-urosepsis were not statistically significant (0.52 ± 1.13 vs. 0.53 ± 0.69, *p* = 0.974). The results were listed in Table 2 and Fig. 1.

**Table 2:** PCT and IL-6

Patient characteristic	EXP group	CON group	*p* values
PCT(preoperative day one)	0.05 ± 0.02	0.05 ± 0.02	0.717
Urosepsis	0.05 ± 0.03	0.05 ± 0.20	0.771
Non-urosepsis	0.05 ± 0.02	0.05 ± 0.02	0.691
PCT(postoperative hour two)	0.14 ± 0.18	0.19 ± 0.23	0.126
Urosepsis	0.39 ± 0.28	0.45 ± 0.33	0.645
Non-urosepsis	0.11 ± 0.13	0.11 ± 0.10	0.884
PCT(postoperative day one)	1.86 ± 4.12	6.85 ± 16.32	0.015
Urosepsis	11.98 ± 4.52	26.57 ± 24.54	0.029
Non-urosepsis	0.52 ± 1.13	0.53 ± 0.69	0.974
IL-6(preoperative day one)	4.43 ± 2.24	5.18 ± 3.39	0.111
Urosepsis	3.92 ± 2.35	5.16 ± 2.28	0.206
Non-urosepsis	4.492 ± 2.24	5.18 ± 3.69	0.204
IL-6(postoperative hour two)	479.76 ± 1160.54	1160.77 ± 1986.13	0.011
Urosepsis	3535.78 ± 891.68	4487.47 ± 1179.18	0.045
Non-urosepsis	75.29 ± 110.81	93.72 ± 167.28	0.469
IL-6(postoperative day one)	43.45 ± 85.31	121.61 ± 209.48	0.005
Urosepsis	236.99 ± 131.91	429.81 ± 232.14	0.031
Non-urosepsis	17.84 ± 21.93	22.76 ± 25.11	0.253

**Fig. 2:**
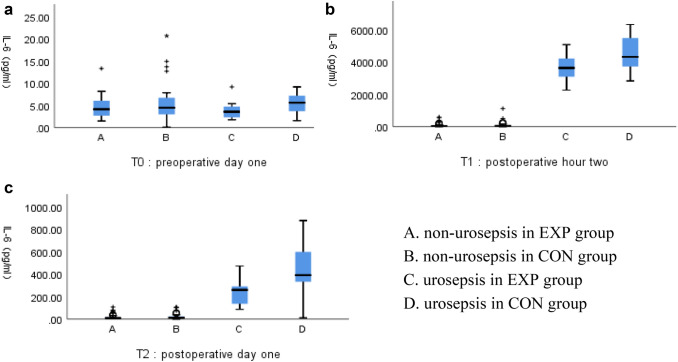
The expression of IL-6 in different groups (A non-urosepsis in EXP group, B non-urosepsis in CON group, C urosepsis in EXP group, D urosepsis in CON group), **a** the expression of IL-6 at preoperative day one, **b** the expression of IL-6 at postperative hour two, **c** the expression of IL-6 at postperative day one

At preoperative day one (T0) univariate analysis showed no significant differences in serum levels of IL-6 (*p* = 0.111) between the EXP group and CON group. At postoperative hour two (T1) the EXP group showed a lower serum level of IL-6 (479.76 ± 1160.54 vs. 1160.77 ± 1986.13, *p* = 0.011) compared with the CON group, the serum level of IL-6 in the EXP group was also lower and statistically significant in patients with postoperative urosepsis (3535.78 ± 891.68 vs. 4487.47 ± 1179.18, *p* = 0.045), but it was not statistically significant in patients with postoperative non-urosepsis (75.29 ± 110.81 vs. 93.72 ± 167.28, *p* = 0.469). At postoperative day one (T2), the EXP group showed a lower serum level of IL-6 (43.45 ± 85.31 vs. 121.61 ± 209.48, *p* = 0.005) compared with the CON group, the serum level of IL-6 in the EXP group was also lower and statistically significant in patients with postoperative urosepsis (236.99 ± 131.91 vs. 429.81 ± 232.14, *p* = 0.031), but it was not statistically significant in patients with postoperative non-urosepsis (17.84 ± 21.93 vs. 22.76 ± 25.11, *p* = 0.253). The results were listed in Table 2 and Fig. 2.

**Fig. 3:**
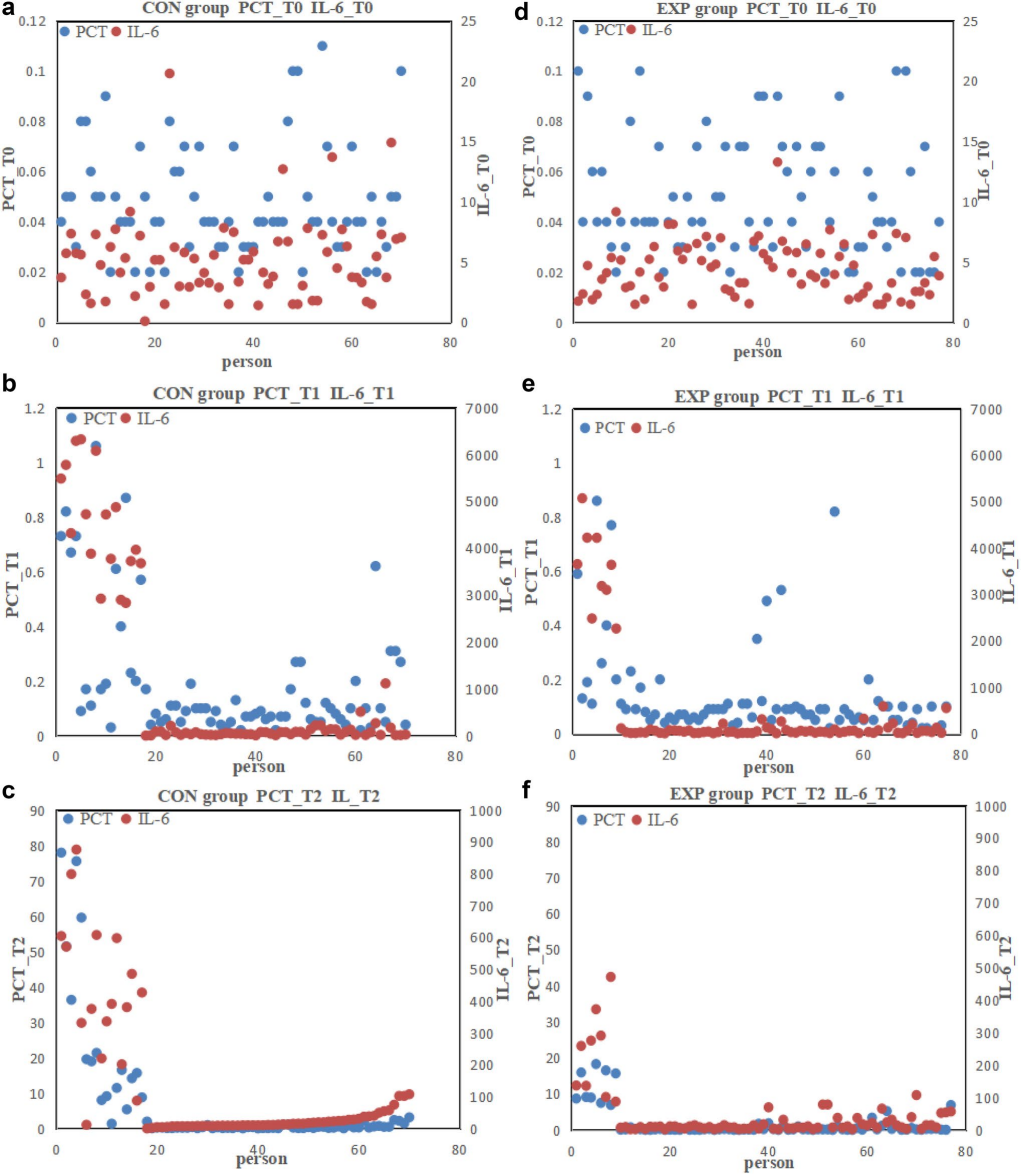
The scatter diagrams of PCT and IL-6 (**a** and **d** the scatter diagram of PCT and IL-6 at preoperative day one in CON group and EXP group, **b** and **e** the Scatter Diagram of PCT and IL-6 at postperative hour two in CON group and EXP group, **c** and **f** the Scatter Diagram of PCT and IL-6 at postperative day one in CON group and EXP group)

PCT and IL-6 are multi-dimensionally displayed by the Radviz in Fig. 3, which can visually display the spatial distribution of PCT and IL-6. At T0, the two features are mixed together, which can not distinguish the corresponding differences more effectively. At T1 and T2, the corresponding spatial structure separations can be seen.

**Fig. 4:**
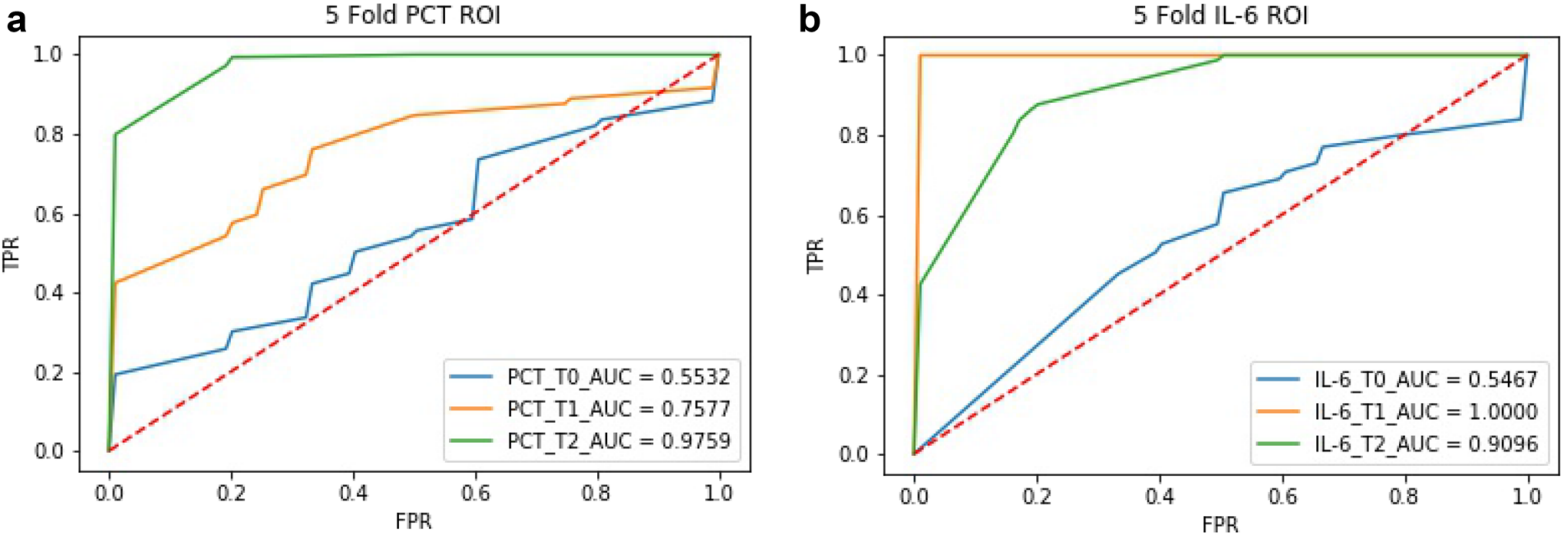
ROCs of identification model of PCT and IL-6 at a different time (T0 preoperative day one, T1 postoperative hour two, T2 postoperative day one), **a** ROC of identification model of PCT, **b** ROC of identification model of IL-6

In order to objectively analyze whether PCT and IL-6 can be used to distinguish the corresponding diseases, we use the traditional machine learning method to carry out the classification validation experiment. According to the conclusion given in Fig. 3, we mix the data of the EXP group and the CON group together, and urosepsis and non-urosepsis are labeled as two types. Then, we divide the training set and test set according to the proportion of 7:3. Random forest is a traditional classification algorithm in machine learning, and we use the method of the random forest to test the data respectively. The result of fivefold cross validation is shown by the ROC curve in Fig. 4. By comparing the results, we find that IL-6 is the most effective classifier at T1 (AUC = 1.00) and PCT is the most effective classifier at T2 (AUC = 1.00). It shows that IL-6 can be used to identify the occurrence of urosepsis earlier than PCT.

## Discussion

Now, PCNL has been regarded as the first-line approach for large, multiple renal calculi, especially for staghorn calculi, and is accepted by more and more urologists. However, postoperative fever with a high incidence rate of 21–32.1% which may progress to potentially life-threatening urosepsis has been a great trouble for both patients and surgeons. In patients diagnosed with urolithiasis, bacteria are often contained not only in urine but also in calculi, including infectious and metabolic calculi [21, 22]. Stone-colonizing bacteria and endotoxin will be released when stones are fragmented during PCNL. Moreover, the intraoperative hydraulic pressure caused by the irrigation fluid in the renal collecting system may result in bacteria and endotoxins translocating to the circulatory system through the renal broken mucous surface, as a result, the postoperative infection eventually follows [23]. To find what can alleviate the infection-related complications is necessary.

The present study showed that the incidence rates of urosepsis and postoperative hospital stays of the EXP group were significantly lower than the CON group as a result of the intraoperative application of dexamethasone and furosemide. Compared with the CON group the lower incidence of severe urosepsis in the EXP group might illustrate that the intraoperative application of dexamethasone and furosemide could alleviate the severity of postoperative urosepsis, although it was not statistically significant perhaps for the small sample size and the low incidence rate of severe urosepsis, which was needed to further study. These results might be attributed to the alleviation of inflammatory reaction caused by dexamethasone and furosemide administration, which was demonstrated in the present study by the resulting lower serum levels of IL-6 and PCT.

As we know, dexamethasone which has long-lasting action (36–48 h), is one kind of glucocorticoid and has a greatly anti-inflammatory, immunosuppressive, and antipyretic effect [24], and furosemide has a diuretic effect which may reduce the absorption of bacteria and endotoxins. There has been no research on the application of dexamethasone and furosemide for postoperative anti-inflammation in patients with PCNL.

Inflammation is a complex interplay of pro-inflammatory and anti-inflammatory reactions accompanying by the massive release of proinflammatory cytokines such as IL-6 [25]. IL-6 is a type of cytokine with various of biology activity and secreted from T lymphocytes, fibroblasts, mononuclear macrophages, etc. when inflammation, necrosis, and neoplasm occur [26]. It reflects an inflammatory state, and plays a crucial role in the inflammatory progression, it is also positively related to the severity of the infection and can be used as a reliable indicator to predict the occurrence of severe infection as a sensitive marker [27]. The present study has found that the postoperative serum levels of IL-6 was significantly lower in patients with urosepsis of the EXP group in comparison with those in the CON group, which represented an alleviated severity of infection and inflammation. PCT, as a 116-amino acid polypeptide, is secreted from neutrophils and parenchymal cells when bacterial infections occur and is a valuable biomarker for early diagnosis of systemic infection, especially for sepsis, and PCT can be up-regulated by bacterial endotoxins and proinflammatory mediators such as IL-6 and tumor necrosis factors and will be down-regulated when infection is controlled, and the concentrations of endotoxins and proinflammatory mediators decreas [28–31]. The value of PCT can represent the severity of infection. The present study found that the postoperative serum levels of PCT were significantly lower at postoperative day one in patients with urosepsis of the EXP group, which represented an alleviated severity of infection and inflammation. The difference of the serum levels of PCT at postoperative hour two between the two groups was not statistically significant, which might be caused by the slowly ascending of the serum levels of PCT within 3 h when infection occurred [32].

During PCNL, stone-colonizing bacteria and endotoxin will be released when stones are fragmented and the intraoperative hydraulic pressure will bring bacteria and endotoxin to the circulatory system through the renal broken mucous surface, and infection and inflammation occur finally. Inflammation is initiated at the injured site by macrophages and mast cells. Macrophages and mast cells release pro-inflammatory mediators, including cytokines, lipid mediators, and bioactive amines. Immediately vasodilation, increased capillary permeability, and leukocyte aggregation happen, which will cause a series of systemic inflammatory responses [33]. Furosemide has a greatly diuretic effect and accelerates urine production, which reduces the absorption of bacteria and endotoxin. As a result, infection and inflammation will be alleviated. Dexamethasone has a great anti-inflammatory and antipyretic effect. It inhibits the release of pro-inflammatory mediators such as cyclooxygenase-2, IL-1, IL-6, and TNF-γ and finally alleviates inflammatory response. A single dose of dexamethasone administration can activate endothelial nitric oxide synthase and alleviate systemic inflammatory response through the nongenomic signaling pathway [34].

Although the anti-inflammatory effect of dexamethasone and furosemide administration perioperatively was significant for patients with urosepsis undergoing PCNL, it was not so obvious in patients with non-urosepsis, and the differences were not statistically significant in the present study. Perhaps the sample size was small, the infection of patients with non-urosepsis was mild, small amounts of inflammatory mediators and bacterial endotoxins were released, thus the application of dexamethasone and furosemide could not make an obvious and statistically significant impact on that in the study, which would be explored in future studies.

There are some limitations of the present study. First, it was a retrospective study, and the application of dexamethasone and furosemide might be influenced by the surgeons’ preference. A prospective study needs to be designed to investigate the application of dexamethasone and furosemide without the influence of the surgeons’ preference. The value of dexamethasone and furosemide can be investigated separately. Second, it was a single-center clinical trial, and the sample size was small which represented a part of the patient population. The results need to be replicated in a multicentric study with different patient populations and large samples. Third, the SOFA criteria will be used to illuminate urosepsis in detail, which is more accurate to predicting clinical outcomes than SIRS and can help to assess the value of the perioperative application of dexamethasone and furosemide [35, 36]. Fourth, the present study merely tested the stationary dose of 10 mg we used conventionally. Therefore, dose-dependent effects required a future study to assess the value of the perioperative application of dexamethasone and furosemide based on individualized drug administration.

In summary, the present study showed that the perioperative application of dexamethasone and furosemide administration during PCNL could alleviate postoperative inflammatory reaction, including reducing the incidence rate of urosepsis and alleviating the severity of postoperative urosepsis, by inhibiting the release of inflammatory mediators and reducing the absorption of bacteria and endotoxin and caused a shorter postoperative hospital stay in patients when postoperative urosepsis occurred. Further researches should be done continuously in the future.
